# Origins and mechanisms leading to aneuploidy in human eggs

**DOI:** 10.1002/pd.5927

**Published:** 2021-03-22

**Authors:** Lena Wartosch, Karen Schindler, Melina Schuh, Jennifer R. Gruhn, Eva R. Hoffmann, Rajiv C. McCoy, Jinchuan Xing

**Affiliations:** ^1^ Department of Meiosis Max Planck Institute for Biophysical Chemistry Göttingen Germany; ^2^ Department of Genetics Rutgers, The State University of New Jersey Piscataway New Jersey USA; ^3^ Human Genetics Institute of New Jersey Rutgers, The State University of New Jersey Piscataway New Jersey USA; ^4^ DNRF Center for Chromosome Stability Department of Cellular and Molecular Medicine Faculty of Health and Medical Sciences University of Copenhagen Denmark; ^5^ Department of Biology Johns Hopkins University Baltimore Maryland USA

## Abstract

The gain or loss of a chromosome—or aneuploidy—acts as one of the major triggers for infertility and pregnancy loss in humans. These chromosomal abnormalities affect more than 40% of eggs in women at both ends of the age spectrum, that is, young girls as well as women of advancing maternal age. Recent studies in human oocytes and embryos using genomics, cytogenetics, and in silico modeling all provide new insight into the rates and potential genetic and cellular factors associated with aneuploidy at varying stages of development. Here, we review recent studies that are shedding light on potential molecular mechanisms of chromosome missegregation in oocytes and embryos across the entire female reproductive life span.

## Key Points

What is known about this topic?

- Aneuploidy is a major cause of preclinical and prenatal fetal losses and congenital disorders in live born.
- Aneuploidy is highly dependent upon maternal age, especially advanced maternal age.
- Aneuploidy is predominantly from the mother.

What does this study add?

- Aneuploidy originates from oocytes and preimplantation embryos.
- Aneuploidy follows a U curve with female age.
- New cellular and molecular mechanisms causing aneuploidies are discussed, including modelling approaches.

## 1 INTRODUCTION

**FIGURE 1:**
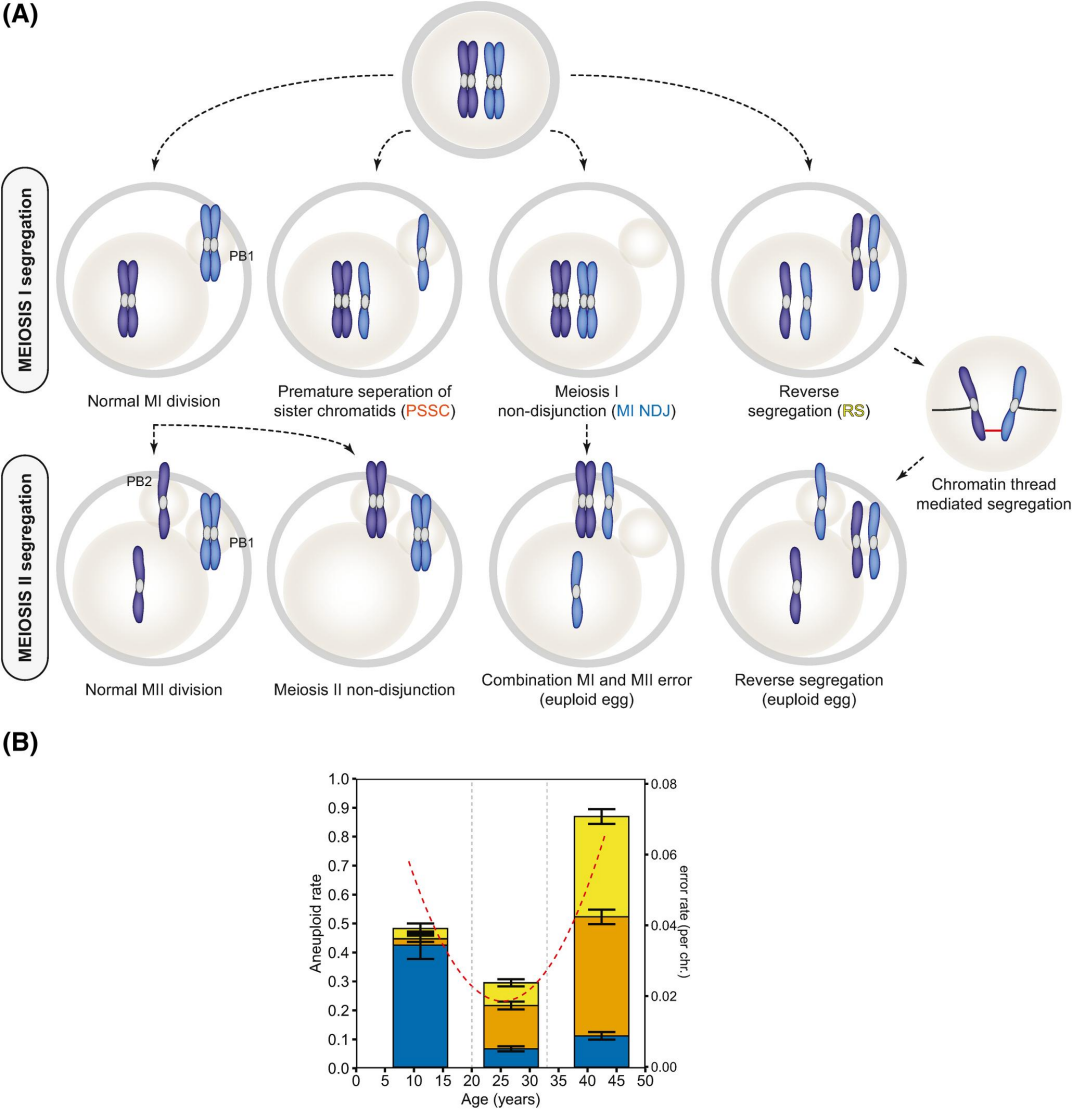
Meiosis I and meiosis II segregation errors and their age dependency in human oocytes. (A) Chromosome segregation patterns in meiosis I and meiosis II. (B) The U‐curve of aneuploidy in human oocytes (dotted red line) is a compilation of all three chromosome missegregation events—MI NDJ (blue), PSSC (orange), and RS (yellow)—and acts in an age dependent manner. MI NDJ, MI non‐disjunction; PSSC, premature separation of sister chromatids; RS, reverse segregation

We have appreciated for a long time that human conceptions are highly error‐prone in terms of whole chromosome gains and losses (aneuploidy; Table 1). Many of these originate in the germline, but there is also a substantial contribution from preimplantation embryos. Germ cells undergo a specialized cell division—meiosis—where a single round of genome duplication is followed by two consecutive chromosomal divisions resulting in haploid gametes (sperm and eggs in human). Thus, upon fertilization, the typical chromosome content is restored with one set contributed by each parent. In human sperm, meiosis lasts about 50–70 days. However, in human oocytes, meiosis is a decades‐long process involving multiple cell‐cycle starts and stops, and it is coupled to acquisition of developmental competence to support fertilization and early embryonic development. In oocyte meiosis, DNA replication and meiotic recombination are completed during fetal development and the cell arrests at the dictyate (diplotene) stage surrounded by supporting mitotic cells. At this stage, homologous chromosomes are tethered together in a bivalent configuration due to crossover recombination between homologous chromosomes and cohesion between sister chromatids. This bivalent configuration has to be maintained for decades until ovulation of the egg, when meiosis I (MI) is completed and homologous chromosomes segregate from each other reducing the genome content by one half. This extended dictyate arrest as well as vulnerable recombination configurations are two major reasons why aneuploidy in human eggs is at least an order of magnitude higher than sperm. The mature ovulated egg arrests at metaphase II and only completes the second meiotic division, where sister chromatids segregate, upon fertilization by sperm (Figure 1A). The embryo continues to develop until the blastocyst stage, when it reaches the uterus, hatches and implants.

**TABLE 1:** Incidence of aneuploidy in the human germline and early development

Genomic alteration	Eggs	Sperm	Preimplantation embryos—cleavage	Preimplantaion embryos—blastocysts	Pregnancy loss	Stillbirths	Live births
Whole chromosome aneuploidy	30% (20%–85% pending age)	2.5% (2.5%–7%)	Up to 70%	56%	50%–60%	6.9%	1:1000
Most common aneuploidies	Young: +1–5; AMA: +13–15; +16; +21; +22	+13; +15; +21; +22; sex chr.	+15; +16; +21; +22	+15; +16; +21; +22	+13; +15; +16; +18; +21; +22	+13; +18; +21; sex chr.	+13; +18; +21; sex chr.
References	1, 2, 3	4, 5, 6	^7^	^8^	^9^	^10^	^11^

Human fetal and adult oocytes have been studied for more than 60 years. Fetal oocytes are obtained from fetal ovaries (Weeks 12–24), whereas adult oocytes were originally obtained from hysterectomies (e.g., Jagiello et al.^12^). The past decades, the success and widespread use of in vitro fertilization (IVF) has made fertility clinics the major source of adult oocytes. More recently, oocytes obtained during ovarian tissue cryopreservation have also been developed as an ex vivo source.^1^, ^13^ The large number of oocytes obtained from different sources have enabled broad conclusions to be reached, including that errors in chromosome segregation resulting in aneuploidy is a general feature of human oocytes and preimplantation embryos. Whereas aneuploidies are highly affected by maternal age, segregation errors during embryonic divisions are independent of maternal age factors. Furthermore, large screening studies of human preimplantation embryos (preimplantation genetic testing for aneuploidies [PGT‐A]) are enabling studies of aneuploidies in thousands of embryos (e.g., Franasiak et al.^14^ and Girardi et al.^8^). Such studies have revealed that meiotic errors are propagated to the blastocyst stage, where they result in vast preclinical as well as clinical losses. In contrast, mitotic errors occurring during the mitotic divisions in preimplantation development appear to be correlated with embryonic or cellular arrest and also give rise to genomically mosaic embryos.^7^, ^15^, ^16^ Below, we review the advances in our understanding of the origins of aneuploidies and the mechanisms that give rise to them.

### 1.1 The U‐curve of aneuploidy and types of meiotic segregation errors in human oocytes

In human oocytes, chromosome segregation errors during the first meiotic division are more prevalent compared to meiosis II (MII) (Table 2 and reviewed by Herbert et al.^17^). Three different abnormal segregation patterns have been found to contribute to the high rate of human aneuploidy: premature separation of sister chromatids (PSSC),^18^ reverse segregation (RS),^2^ and meiosis I non‐disjunction (MI NDJ).^19^ In a recent study by Gruhn et al.,^1^ missegregation events were identified in human oocytes spanning the entire reproductive life span (9–43 years) and were found to follow a U‐curve (Figure 1A, dotted red line) formed by a compilation of all three error patterns (Figure 1A, bar graph). More importantly, these error patterns influence aneuploidy levels in not only an age‐dependent, but also a chromosome‐dependent manner (Table 2).

**TABLE 2:** Incidence of MI versus MII errors in human oocytes by chromosome (data from Gruhn et al.^1^ and Zielinska et al.^23^)

Chr.	*N*	MI error (%)	MII error (%)
1	9	77.8	22.2
2	3	100	0
3	3	100	0
4	7	71.4	28.6
5	5	60.0	40.0
6	4	100	0.0
7	2	50.0	50.0
8	6	100	0
9	0	0	0
10	6	100	0
11	2	100	0
12	1	100	0
13	7	100	0
14	3	100	0
15	3	100	0
16	12	83.3	16.7
17	2	100	0
18	4	75.0	25.0
19	5	100	0
20	6	66.7	33.3
21	6	83.3	16.7
22	9	77.8	22.2
*X*	4	100	0
Acrocentrics	28	89.3	10.7
Non‐acrocentrics	81	85.2	14.8

Abbreviation: MI, meiosis I; MII, meiosis II.

**FIGURE 2:**
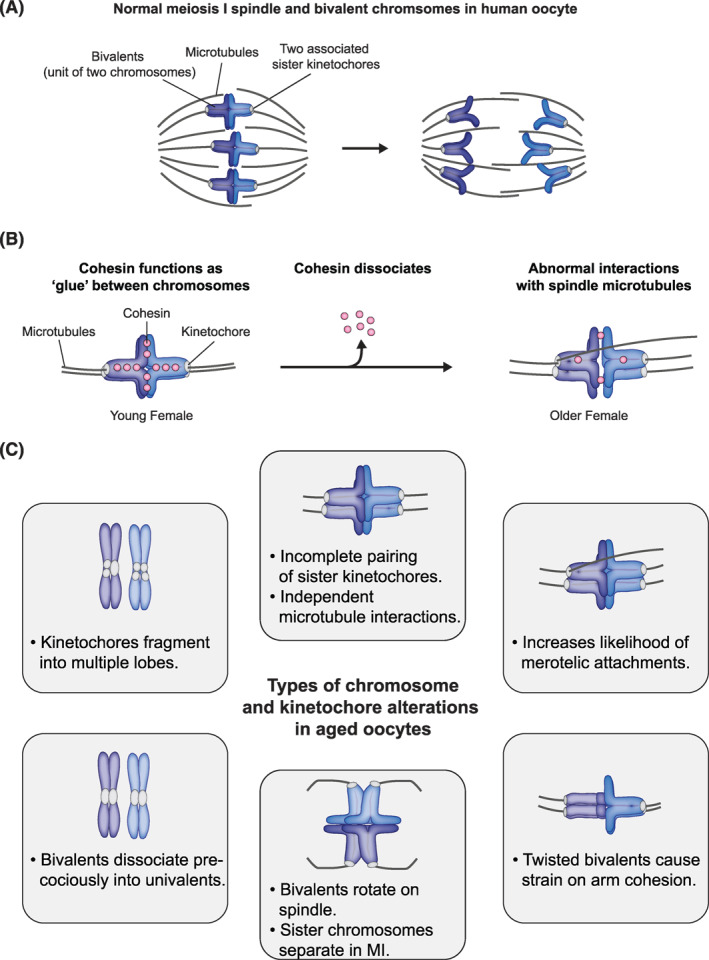
Structural changes and alternative alignments of chromosomes during meiosis I. (A) During MI, bivalents align at the metaphase plate of the meiotic spindle before chromosomes are segregated. (B) Cohesin is lost from chromosomes with advancing female age. (C) Schematic illustrating alterations in the architecture of chromosome bivalents with advancing maternal age. MI, meiosis I

PSSC was originally identified as the most common segregation error type in oocytes, primarily due to its strong positive correlation with maternal age.^20^, ^21^ PSSC, or the separation of one set of sister chromatids at MI instead of MII, leads to a 3:1 division of chromatids and the formation of two aneuploid daughter cells (Figure 1B). This segregation pattern increases linearly with age and has led to the hypothesis that premature loss of centromeric cohesion during the prolonged dictyate arrest and abnormal kinetochore attachments may directly lead to PSSC events in oocytes as maternal age increases.^22^, ^23^, ^24^, ^25^ In cases where more extreme cohesion loss occurs (i.e., weakening of both centromeric and arm cohesion in MI) or where homologous chromosomes failed to crossover, an RS event can arise where both sets of sister chromatids separate prematurely in MI.^1^, ^2^ RS was discovered to occur at an incidence nearly 100× higher than expected from two individual PSSC events,^1^, ^2^ suggesting a distinct origin from PSSC. Indeed, RS shows a dramatic increase between the mid‐ and advance maternal age groups consistent with more extensive cohesion loss along entire chromosome arms or on both sets of centromeres. Both PSSC and RS were found to primarily affect the acrocentric chromosomes (chr. 13‐15 21 & 22), thus suggesting a potential correlation between cohesion loss and aneuploidy incidence (Figure 2, discussed below).^1^

Recently, however, a third pattern has re‐emerged as major error type through the inclusion of a wider age range in oocyte aneuploidy analysis.^1^ MI NDJ, where the homologous chromosomes fail to separate and all four sister chromatids enter into one daughter cell (4:0), was found to be extremely prevalent at younger maternal ages (<20). Further analysis showed that not only does MI NDJ occur primarily in oocytes from young women, but the chromosomes impacted by this error type are predominantly the largest chromosomes (chr. 1–5). These chromosomes show a lower likelihood of being aneuploid in embryos^28^, ^29^ and clinically recognized pregnancies^26^, ^27^; therefore, suggesting that many of the rates seen at later developmental stages are greatly underestimating the prevalence of aneuploidy along the entire age spectrum.

Segregation errors can also occur in MII after fertilization (Table 2); however, these errors can have either a detrimental or a “beneficial” effect.^30^, ^31^ In cases where MI segregation occurs normally, the sister chromatids may undergo meiosis II non‐disjunction (MII NDJ) and both chromatids are pulled to a single daughter cell resulting in an aneuploid conception. However, there are cases where a combination of MI and MII errors result in a normal euploid oocyte^30^ (Figure 1A). In addition, there is evidence that in 75% of RS events in MI—where the chromatids have no physical connection and should segregate independently—they are able to segregate correctly.^1^, ^2^ This “correct” segregation of two non‐sister chromatids in MII may be mediated by chromatin threads that connect chromosomes and were discovered recently^1^ (Figure 2). Thus the impact on aneuploidy in conceptuses depends on the type of MI error and the incidence of MII error—for RS aneuploidy risk in the embryo is lower compared to PSSC (50% cause aneuploidy in embryos) and MI NDJ (all cause aneuploidy in embryos, except if a second error occurs in MII, see below).

## 2 UNDERSTANDING SEGREGATION ERRORS LEADING TO ANEUPLOIDY WITH MATHEMATICAL MODELLING

One limitation with direct studies of human eggs is obtaining sufficient numbers. With the improved understanding of chromosome segregation errors in human oocytes, however, it has been possible to apply mathematical modeling to expand our knowledge by analyzing large datasets generated from PGT‐A of human embryos. One limitation with direct observations in human preimplantation embryos as well as conceptuses is that only the final meiosis product (the egg) is available. This makes trisomic conceptions due to MI NDJ, PSSC, and RS indistinguishable, since any of these errors would result in a conception with two non‐homologous chromosomes originating from the egg.

In a recent study,^31^ the authors constructed a mathematical model for different types of meiotic errors. Using this model, the authors formulated the proportion of resulting zygotes of chromosome segregation errors (e.g., normal, single trisomy, double trisomy, etc.) depending on the rates of MI NDJ, PSSC, and MII NDJ. By fitting the model to observed aneuploidy data from 11,157 embryos (based on PGT‐A), the best‐fitting models revealed several biological insights of female aneuploidy. For example, eggs with MI NDJ errors are more likely to have errors in MII, suggesting an association of the error mechanisms in the two meiotic stages. As observed in the several previous studies from the IVF field (e.g., Magli et al.^30^), the model suggests that oocytes with certain MI and/or MII errors can result in a euploid embryo. For example, a combination of MI NDJ and MII NDJ can result in a euploid embryo (Figure 1A), although these account for fewer than 1% of embryos. As the meiotic error rates increase with maternal age, the proportion of euploid embryos resulting from meiotic errors increase with maternal age as well. The most common type is PSSC followed by a normal MII segregation, which has a 50% chance of resulting in a euploid embryo. In addition, the authors demonstrated the potential clinical utility of the modeling approach. By simulations based on the best‐fitting model, they determined the possibility of having at least one euploid embryo given the patient's age and the number of embryos tested. This information can potentially help clinicians and patients to estimate the expected number of IVF cycles needed to obtain a euploid conception without relying on having a diagnosis, such as specific genetic mutations that predispose to aneuploidy.

### 2.1 Mechanisms of aneuploidy formation: establishment and maintenance of bivalents

The segregation of entire chromosomes during MI relies on the formation of bivalents, which consist of two joined homologous chromosomes (Figure 2A). Both their establishment and their maintenance until the first meiotic division contribute towards the high incidence of aneuploidy in human eggs. Bivalents are formed during fetal development in meiotic prophase I via meiotic crossover recombination and then have to be maintained for decades, until ovulation of the egg. The sites of genetic exchange bring homologous chromosomes together and their cytological manifestation (“chiasma”) are visible due to the cohesion between the distal arm regions of the sister chromatids. Cohesion in centromeres and pericentromeres between sister chromatids is important for their co‐orientation such that sister chromatids segregate to one pole at MI.

Aberrant crossover recombination patterns—such as poor placement of exchanges or exchange‐less chromosomes—have been identified as important factors in the formation of aneuploidy in human eggs and sperm.^2^, ^3^, ^4^, ^5^, ^6^, ^32^, ^33^ Up to 7% of human fetal oocytes contain at least one exchange‐less chromosome, which predominantly affect the smallest chromosomes 21 and 22^33^ and “programmed” inefficiency in human eggs, but not sperm, has been proposed to contribute to this high incidence.^34^ Exchange‐less chromosomes may predominantly undergo RS, bi‐orienting their kinetochores to opposite spindle poles during MI.^1^, ^23^, ^35^

The maintenance and integrity of the bivalent during the decades‐long dictyate arrest relies on a specialized meiotic REC8‐cohesin complex. Cohesin in the chromosome arm region distal to the recombination site holds the bivalents together until ovulation and the onset of anaphase I, when cleavage of REC8‐cohesin in the arm region allows the chromosomes to segregate.^36^, ^37^ Decreased or diminishing cohesin protein levels is hypothesized as a key “hit” leading to the age dependent increase in aneuploidy. Work in mice suggests that the REC8‐cohesin complex is loaded prior to meiotic recombination, with no substantial turnover or replenishment throughout the prolonged arrest period.^38^, ^39^ As mice age, the cohesin complex is gradually lost from chromosomes and consequently, the bivalent architecture changes (Figure 2B,C).^39^, ^40^, ^41^, ^42^ The homologous chromosomes within a bivalent become separated by prominent gaps, and sometimes dissociate into univalents.^43^, ^44^ Consistent with defects in cohesin being sufficient to cause bivalent erosion with age, a meiosis‐specific cohesin component, *SMC1β*, is haploinsufficient in mouse. Adult oocytes from *SMC1β* heterozygous females show age‐dependent loss of bivalents and elevated aneuploidy rates.^45^ Whether cohesin is lost in human oocytes is still unclear as studies investigating levels of individual cohesin subunits in oocytes of different ages come to different conclusions.^46^, ^47^ However, it is clear that the architecture of bivalents in human oocytes changes dramatically as women get older.^23^, ^24^, ^35^, ^43^ Bivalents become separated by gaps and dissociate into univalents, similar to what happens in mouse oocytes. This is particularly relevant for the smaller chromosomes 21 and 22 that have less arm cohesion than larger chromosomes and show elevated frequency of univalents in women of advanced maternal age.^48^

#### 2.1.1 Lack of sister kinetochore co‐orientation in meiosis I is a common source of missegregation

A dogma in the meiosis field is that sister kinetochores are fused during MI to permit their co‐segregation at anaphase I, resulting in both sister chromatids of the homolog moving towards the same spindle pole thereby completing the reductional division of meiosis.^29^, ^49^ The fusion of the sister kinetochores relies on REC8‐cohesin complexes in the centromeric and pericentromeric regions, similar to arm regions (Figure 2B).^50^, ^51^ REC8‐cohesin in peri‐ and centromeric regions is protected from cleavage during anaphase I by shugoshin proteins.^52^ This protection is essential to maintain the association of sister chromatids within a chromosome at the MI to MII transition.

Interestingly, human oocytes challenge the dogma of fused sister kinetochores. Although aging also affects the fusion of sister kinetochores, young women already show a substantial fraction of kinetochores separated by large gaps.^23^, ^24^ These split sister kinetochores can interact with spindle microtubules independently and hence do not function as a single unit during the first meiotic division. Notably, the separation of sister kinetochores increases with female age. This separation of sister kinetochores favors several incorrect types of chromosome attachments to the spindle in MI. Most prominently, it favors the formation of merotelic attachments, where a kinetochore is linked to both spindle poles, instead of being linked to a single spindle pole only.^23^ Moreover, it allows bivalents to rotate on the spindle, resulting in a configuration where sister kinetochores are facing opposite spindle poles like in mitosis, instead of facing to the same spindle pole. These inverted bivalents may lead to improper separation of sister chromatids during anaphase I, which may result in PSSC or RS.^2^, ^29^

The separation of sister kinetochores can also explain why prematurely dissociated univalents often align on the MI spindle like mitotic chromosomes, with sister kinetochores orienting towards opposite spindle poles.^23^, ^43^, ^53^, ^54^ Segregation of these univalents into sister chromatids during MI could be another mechanism that contributes to the RS pattern. Moreover, having four instead of two microtubule attachment sites can further cause twisting of bivalents along their axis.^23^ Such twisting is likely to put additional force on chromosome arm cohesion.^23^ The separation of premature separation of bivalents into univalents, as well as splitting of sister kinetochores might be further exacerbated by multidirectional pulling of spindle microtubules during the prolonged spindle assembly process in human oocytes.^55^

Recent studies further revealed that centromeres and kinetochores themselves change in aged oocytes of different mammalian species, including humans. The core centromere protein Cenp‐A becomes depleted from centromeres.^35^, ^56^ Moreover, centromeric chromatin decompacts. Kinetochores built on loosened centromeric chromatin get destabilized and fragment into multiple lobes.^35^ Such fragmented kinetochores are often merotelically attached on the metaphase II spindle, and may thereby contribute to aneuploidy. Fragmented kinetochores are further characterized by reduced levels of key components of the inner and outer kinetochore regions, which may further compromise kinetochore function.^35^

#### 2.1.2 The spindle assembly checkpoint, perturbed protein homeostasis, and differential mRNA expression are potential mechanisms resulting in human aneuploidy

Model organisms, such as mice, have provided much of our knowledge on genes and pathways that are important for protecting mammalian gamete euploidy.^57^ Upon resumption of meiosis in the adult ovary the chromosomes are condensed, the spindles are made, and kinetochore attachments are formed. Importantly, at this stage the spindle assembly checkpoint (SAC), a complex mechanism that integrates the attachment status of the kinetochore with spindle microtubules, must be satisfied. If anaphase onset occurs when a kinetochore is unattached, an MI bivalent will fail to disjoin. Unattached kinetochores arise either from faulty spindle building, which is common in human oocytes,^55^ or from Aurora kinase activity sensing an improper attachment and triggering microtubule depolymerization.^58^ When kinetochores are unoccupied, MPS1 kinase initiates the SAC response and triggers recruitment of scaffold proteins that assemble the mitotic checkpoint complex (MCC). The MCC diffuses and sequesters CDC20, thereby preventing anaphase promoting complex/cyclosome (APC/C) activation and arrests the cell cycle.^59^, ^60^ In somatic cells, one unoccupied kinetochore will trigger the SAC,^61^ but in mammalian oocytes, the SAC is more permissive, and can fail to prevent anaphase even in the presence of several misaligned chromosomes.^62^, ^63^, ^64^, ^65^, ^66^ A weaker SAC has been proposed to predispose oocytes to MI chromosome segregation errors.^62^, ^67^

Another pathway that appears to sensitize oocytes to chromosome missegregation is gene expression and control through regulated translation.^68^ Oocytes complete MI and MII in the absence of transcription and instead mount a highly regulated burst of translation during pro‐metaphase I. This strategy requires proper storage of repressed maternal transcripts during oocyte growth and the prophase I arrest.^69^ Upon meiotic resumption, oocytes must switch off the repression and activate their translation. Repressed messages are enriched for cell‐cycle regulators and transcriptional and epigenetic machinery encoding proteins that are required later in meiosis, fertilization, and/or embryogenesis. Regulation of translation is therefore critical to producing proteins essential for accurate chromosome segregation. Because perturbations in protein homeostasis (i.e., proteostasis) are associated with the aging process,^70^, ^71^ it is tempting to speculate that oocytes from women of advanced maternal age are uniquely sensitive to abnormal protein expression levels that could make them more prone to aneuploidy. This is further supported by findings that mRNA expression of cell cycle and DNA repair genes are differentially downregulated in aged human eggs.^72^

## 3 GENETIC CONTRIBUTIONS AND PATHWAYS ASSOCIATED WITH ANEUPLOIDY RISK

The parental genetic contribution to aneuploid conception risk is a topic of long‐standing interest in human reproductive biology. Although increased risk of aneuploidy is strongly correlated with increasing maternal age, significant variation exists in aneuploid conception rates of IVF patients without any reproductive pathology at any given age.^73^, ^74^, ^75^, ^76^, ^77^ Indeed, some of the first genetic surveys of human preimplantation embryos noted that certain patients appeared to be predisposed to generating embryos with complex forms of mosaic aneuploidy (i.e., “chaotic mosaicism”), independent of maternal age.^78^ Similar observations have been noted with respect to oocyte and sperm aneuploidies of meiotic origin, including in recent studies.^4^, ^76^ These observations point to the possibility that inherited genetic variation influences the fidelity of meiosis and/or early embryonic mitosis, the latter controlled by maternal gene products deposited in the oocyte.

It is worth noting that no formal estimate of the heritability of aneuploidy risk has yet been achieved, which likely owes to the challenge of defining and measuring this phenotype (generally based on rates from clinical programs of PGT‐A) in a sufficiently large cohort for quantitative genetic analysis. Such estimates generally require approximately 5000–10,000 samples to achieve substantial power for detecting modest effects, and may require even more if the true heritability is low or the phenotype is measured with error.^79^ Nevertheless, the discovery of aneuploidy‐associated variation represents an outstanding goal in human reproductive genetics, with the potential to reveal mechanisms of aneuploidy formation. Even without such mechanistic knowledge, the genetic associations also hold promise for improving precision in prediction of aneuploidy risk when combined with known covariates such as maternal age. Such comprehensive risk assessment may be useful for informing reproductive decisions, in the context of IVF or natural conception.

Genome‐wide association studies (GWAS) offer a powerful approach for discovering common genetic variation influencing complex traits such as risk of aneuploid conception. This phenotype may be quantified as a proportion of aneuploid embryos produced in an IVF cycle, as measured by PGT‐A. Using parental genotype data (*N*_maternal_ = ∼5000) from single‐nucleotide polymorphism (SNP) based PGT‐A, McCoy et al.^80^ discovered a maternal haplotype spanning the centrosomal regulator polo‐like kinase 4 (*PLK*4) that is associated with complex aneuploidy of mitotic origin in day‐3 embryo biopsies. Through follow‐up analysis and intersection with time‐lapse data, they later proposed that this association is driven by a mechanism of tripolar mitosis, whereby diploid cells segregate their chromosomes on a tripolar spindle, leading to massive chromosome loss.^81^, ^82^ Interestingly, this signature is rare in data from the blastocyst stage of development at day 5, and patients with the high‐risk genotype have fewer embryos that achieve blastocyst formation—together suggesting that complex mosaic aneuploidy compromises development to the blastocyst stage.^7^, ^82^, ^83^ Yet depending on their timing of occurrence, even these complex forms of mosaicism may not preclude development, and indeed may be preferentially excluded from the embryo during the process of blastocyst formation.^84^, ^85^ While explaining a small fraction (∼1%) of the total variance in aneuploidy risk, these examples demonstrate how GWAS can be valuable for generating hypotheses about mechanisms of aneuploidy formation.

Only a small proportion of aneuploidies are compatible with live birth, and those that result in genetic disorders such as Down Syndrome, merit additional focus. Seeking to understand the potential maternal genetic contributions to the origins of trisomy 21, Chernus et al.^86^ conducted a candidate gene association study for variants influencing trisomy 21 of MI and MII origin (*N*_maternal_ = ∼700). Using SNP array genotyping data from parents and children with trisomy 21, they classified meiotic errors based on heterozygosity of pericentromeric markers, then contrasted maternal genotypes with those of the fathers, which served as a natural internal control. Genome‐wide analyses revealed no variants reaching genome‐wide significance (typically defined as *p* < 5 × 10^−8^), but several of the top suggestive associations occurred within or near genes with known meiotic functions, including *AURKC*, an Aurora kinase whose dysregulation is known to induce aneuploidy in mouse models.^87^, ^88^ Further focus on candidate meiosis‐related genes revealed additional potential associations for validation and follow‐up study.

In contrast to association studies, which are generally based on SNP array genotyping at sites of known variation in the human population, the discovery of rare variants that influence aneuploidy risk requires alternative approaches, such as whole genome or targeted sequencing, as well as functional validation in model organisms or human cell lines. For example, targeted sequencing of candidate genes in patients experiencing recurrent IVF failure identified loss‐of‐function variants in a primate‐specific tubulin b class VIII (*TUBB*8),^89^, ^90^, ^91^ PAT1 homolog 2 (*PATL2*),^92^, ^93^, ^94^, ^95^, ^96^, ^97^ and WEE2 oocyte meiosis inhibiting kinase (*WEE2*)^98^, ^99^, ^100^, ^101^, ^102^ that may predispose women to a higher incidence of oocyte and embryonic aneuploidy at younger‐than‐average ages. The products of these genes are required for essential steps in oocyte development and meiosis. On the other hand, gene variants may exist that protect euploidy and extend a woman's reproductive life span. One study identified a variant in Aurora kinase B, a protein involved in the SAC, which conferred a protective advantage in an older (39 years) IVF patient.^77^

As the sequencing cost decreases, whole exome and whole genome sequencing are increasingly becoming more feasible. Comparing to the candidate gene approach, whole exome/genome studies is not limited to predetermined candidate genes and have the potential of discovering novel genetic mechanisms for aneuploidy. For example, a recent study applied exome sequencing to compare patients with high and low frequencies of aneuploid blastocysts to identify genetic factors responsible for chromosome segregation errors.^76^ The power of this approach is the specific aneuploid embryo phenotype and the ability to group patients into extreme phenotype categories. The analysis of nearly 100 exomes of women who produced greater than 50% aneuploid blastocysts during IVF‐revealed variant enrichment in genes that function in biological processes such as “centriole,” “DNA repair,” and “damaged DNA binding.” In vitro assessment of one of the high‐ranking “centriole” variants (CEP120 p.Arg947His) using mouse oocytes revealed that women who are heterozygous for this allele may produce aneuploid eggs because of inefficient microtubule nucleation.^76^ Despite studies like these, a comprehensive understanding of all genes contributing to embryonic aneuploidy and the relative contributions of common and rare variation to aneuploidy phenotypes is still lacking.

## 4 CONCLUSIONS

Our current understanding of human aneuploidies has increased dramatically the past decade, facilitated by large studies of human eggs, sperm, and embryos. Research in the areas of meiotic recombination, chromosome cohesion weakening, abnormal kinetochore structures, extended effects of maternal age, and genetic contributions to aneuploidy risk, are all moving us closer to understanding the origins of aneuploidy and therefore the potential for future clinical interventions. Once further molecular mechanisms associated with individual targets are identified, the field may improve diagnoses and genetic screening programs, as well as increase the efficacy of conceptions by developing interventions to reduce aneuploidy rates. Improved understanding of genomic mosaicism in preimplantation embryos, especially in placental lineages, may also contribute to advances in prenatal diagnostics.

## CONFLICT OF INTERESTS

The authors declare that there are no conflict of interests.
